# Congenital cataract: a guide to genetic and clinical management

**DOI:** 10.1177/2633004020938061

**Published:** 2020-07-22

**Authors:** Suzannah J. Bell, Ngozi Oluonye, Philippa Harding, Mariya Moosajee

**Affiliations:** Department of Genetics, Moorfields Eye Hospital, London, UK; Department of Genetics, Moorfields Eye Hospital, London, UK; Department of Ophthalmology, Great Ormond Street Hospital for Children, London, UK; UCL Institute of Ophthalmology, London, UK; UCL Institute of Ophthalmology 11-43 Bath Street London EC1V 9EL, UK; Department of Genetics, Moorfields Eye Hospital, London, UK; Department of Ophthalmology, Great Ormond Street Hospital for Children, London, UK

**Keywords:** cataract, congenital cataract, genetics, inherited cataract, paediatric ophthalmology

## Abstract

Worldwide 20,000–40,000 children with congenital or childhood cataract are born every year with varying degrees and patterns of lens opacification with a broad aetiology. In most cases of bilateral cataract, a causative genetic mutation can be identified, with autosomal dominant inheritance being most common in 44% of cases. Variants in genes involve lens-specific proteins or those that regulate eye development, thus giving rise to other associated ocular abnormalities. Approximately 15% of cases have systemic features, hence paediatric input is essential to minimise comorbidities and support overall development of children at high risk of visual impairment. In some metabolic conditions, congenital cataract may be the presenting sign, and therefore prompt diagnosis is important where there is an available treatment. Multidisciplinary management of children is essential, including ophthalmic surgeons, orthoptists, paediatricians, geneticists and genetic counsellors, and should extend beyond the medical team to include school and local paediatric visual support services. Early surgery and close follow up in ophthalmology is important to optimise visual potential and prevent amblyopia. Routine genetic testing is essential for the complete clinical management of patients, with next-generation sequencing of 115 genes shown to expedite molecular diagnosis, streamline care pathways and inform genetic counselling and reproductive options for the future.

**Lay abstract:**

**Childhood cataract: how to manage patients**

Cataract is a clouding of the lens in the eye. Cataract occurring in children has many different causes, which may include infections passed from mother to child during pregnancy, trauma, medications and exposure to radiation. In most cases of cataract occurring in both eyes, a genetic cause can be found which may be inherited from parents or occur sporadically in the developing baby itself while in the womb. Cataracts may occur on their own, with other eye conditions or be present with other disorders in the body as part of a syndrome. Genetic testing is important for all children with cataract as it can provide valuable information about cause, inheritance and risk to further children and signpost any other features of the disease in the rest of the body, permitting the assembly of the correct multidisciplinary care team. Genetic testing currently involves screening for mutations in 115 genes already known to cause cataract and has been shown to expedite diagnosis and help better manage children. Genetic counselling services can support families in understanding their diagnosis and inform future family planning. In order to optimise vision, early surgery for cataract in children is important. This is because the brain is still developing and an unobstructed pathway for light to reach the back of the eye is required for normal visual development. Any obstruction (such as cataract) if left untreated may lead to permanent sight impairment or blindness, even if it is removed later. A multidisciplinary team involved in the care of a child with cataract should include ophthalmic surgeons, orthoptists, paediatricians, geneticists and genetic counsellors, and should extend beyond the medical team to include school and local child visual support services. They will help to diagnose and manage systemic conditions, optimise vision potential and help patients and their families access best supportive care.

## Introduction

Worldwide, 20,000–40,000 children with congenital or childhood cataract are born every year, and there are an estimated 200,000 children blind from bilateral cataract.^1^ In the UK, childhood cataract affects 2.5–3.5 per 10,000 children, with most occurring within the first year of life.^2^ Genetic mutations account for the majority of cases of bilateral cataract, and the most frequent mode of inheritance is autosomal dominant seen in 44% of families.^3^ Important environmental factors to consider include congenital infections such as toxoplasma, syphilis, varicella zoster, parvovirus B19, coxsackievirus, rubella, cytomegalovirus (CMV) and herpes simplex virus I and II (TORCH). Trauma and iatrogenic causes such as medications and radiation exposure are also relevant but rare in this age group. Inherited congenital cataract may occur in isolation (70%), with other ocular abnormalities (complex) (15%) or form part of a syndrome (15%).^4^ Because a wide range of conditions is associated with congenital cataract, often many investigations are performed to identify an underlying cause. There is variability in the investigative pathways of paediatric cataract patients, with some not undergoing any testing despite a family history, or inappropriate and inefficient use of screening tests.^5^ Implementation of genetic testing using next-generation sequencing has provided evidence of streamlining this process.^6^

Surgery for paediatric cataract has many considerations and early interdisciplinary management is important for good long-term visual outcomes. General paediatric involvement is essential to aid diagnosis and management of children presenting with systemic features and in the management of vision in the developing child as well as supporting families in challenges that may present throughout childhood. There is often significant responsibility placed on parents who have a critical role in adherence to intensive treatment – for example, with patching or frequent administration of eye drops post-operatively and multiple hospital visits.

## Aetiology and classification

**Figure 1.**
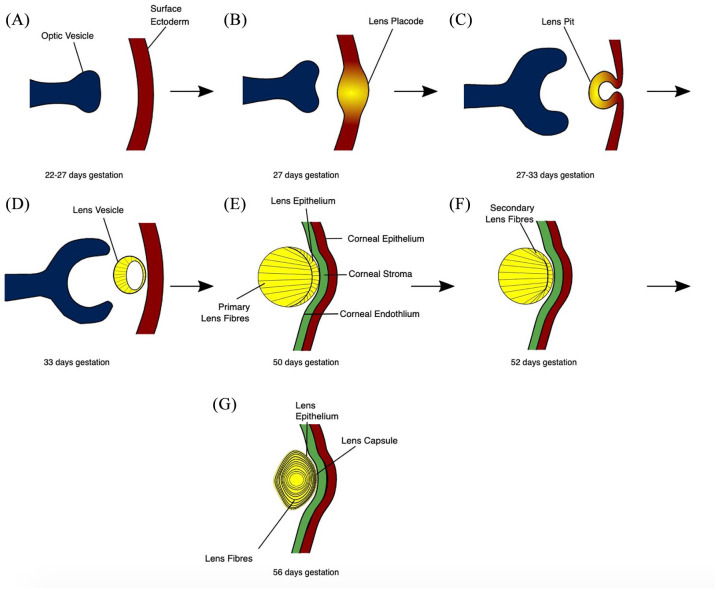
The stages of human lens development. (a) The preplacodal region develops within the surface ectoderm overlying the optic pit/groove. (b) Signalling from the optic vesicle stimulates the preplacodal region to thicken to form the lens placode. (c) Cell proliferation at the lens placode, in addition to morphological changes including apical constriction, result in the formation of a lens pit. (d) Detachment of the lens vesicle from the surface ectoderm, which is rebuilt to later form the corneal epithelium. (e) Posterior epithelial cells elongate anteriorly to occlude the lumen of the lens vesicle. Maturation of the cornea into the epithelium, stroma and endothelium. (f) Epithelial cells at the equatorial regions form secondary lens fibre cells, which elongate anteriorly and posteriorly, encircling the primary lens fibres.

The human lens is formed *in utero* from surface ectodermal cells, which thicken to form the lens placode and then invaginate and pinch off to form the lens vesicle by day 33 of gestation.^7,8^ The posterior epithelial cells elongate anteriorly to occlude the lumen of the vesicle and form the primary lens fibres. Only the anterior epithelial cells at the equatorial regions of the lens divide to form secondary lens fibres, and this continues throughout life (Figure 1).^9^

**Figure 2.**
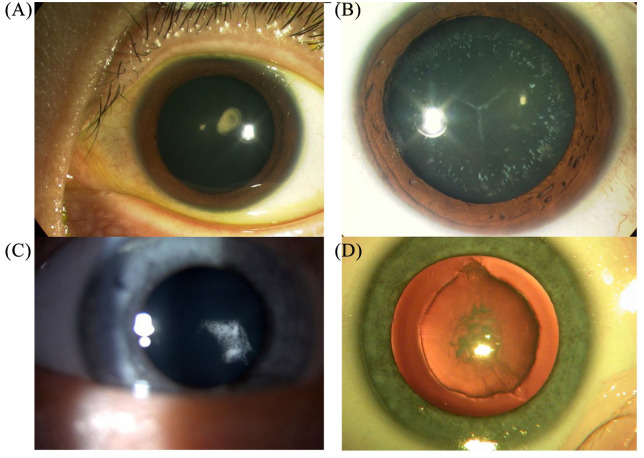
Slit-lamp presentation of inherited cataract phenotypes: (a) anterior polar cataract; (b) blue dot (cerulean) cataract; (c) posterior subcapsular cataract; (d) nuclear cataract (retroillumination, kindly donated by Professor Ian Christopher Lloyd).

Cataract develops due to disruption of the normal lens protein structure or function, resulting in opacity. This may occur as a result of stressors applied to lens proteins including those acquired *in utero* or during childhood. However, a significant proportion is due to mutations in the genes encoding lens proteins that directly affect their role within the lens.^8^ Various congenital cataract morphology have been described, including nuclear, posterior/anterior polar, pulverulent lamellar, posterior subcapsular, cerulean or blue dot cataract (Figure 2).

Cataract can be categorised according to age of presentation. True congenital cataract presents at birth, while cataract that develops later in childhood is described in various ways (e.g. developmental, infantile, juvenile) with different criteria for inclusion in these classifications.^10–12^ Categorisation in this way can be challenging, as cataract may exist at birth but not be identified until later, which is important in countries where delayed presentation is an issue.^13^

### Inherited cataract

The proportion of cataract due to genetic mutations is likely higher than we think because many patients are not tested and there are more cataract causing genes to be discovered. It is possible to identify a genetic mutation in up to 90% of cases of bilateral cataract with current genetic testing.^3^ There is substantial genetic and phenotypic heterogeneity with significant intra- and inter-familial variability. Mutations in genes encoding lens proteins can also demonstrate pleiotropic effects – for example, *NHS* gene, which causes Nance–Horan syndrome has a complex pattern of temporally and spatially regulated gene expression involving the development of ocular, craniofacial and neural tissue.^14,15^ For some cataract-associated genes, the resulting phenotype can vary widely depending on the localisation of the respective nucleotide change. The *LSS* gene encodes lanosterol synthase, which is an enzyme involved in the biosynthesis of cholesterol in the body. Romano *et al.* suggested that autosomal recessive mutations occurring towards the N-terminus of this gene are associated with hair loss and those at the C-terminus are associated with lens defects.^16^ This theory is consolidated by Chen *et al*., who reported a mixed phenotype in an individual with baldness, absent eyebrows and congenital cataracts with two heterozygous mutations occurring in *LSS* c.1025T>G, p.(Ile342Ser) and c.1887G>T, p.(Trp629Cys) near the N- and C-terminal regions respectively.^16,17^

#### Non-syndromic congenital cataract

The main lens proteins associated with congenital cataract include crystallins, membrane proteins, cytoskeletal structural proteins and transcription factors. Some of these genes are also involved in the development of other ocular structures; hence in approximately 15% of cases mutations can lead to associated eye abnormalities.^11^

##### Crystallins

α, β and γ-crystallins are the main structural refractive protein of the human lens; thus mutations in this family of genes result in opacity. In addition, α-crystallins behave like molecular chaperones, where they assist in the folding or unfolding of other damaged or denatured proteins such as β and γ-crystallins, and can inhibit apoptosis.^18^ These functions are critical for maintaining lens transparency. Approximately 50% of non-syndromic hereditary cataract are due to mutations in genes coding for crystallin proteins with over 100 mutations, mostly missense, encoding 12 different human α, β and γ-crystallin genes (Table 1).^19^

**Table 1.** Genes associated with isolated cataract. Detailed phenotype data sourced from Genomics England PanelApp and OMIM cataract databases and associated relevant publications.

Gene name/locus	Gene/locus MIM number	Inheritance	Disease MIM number	Disease name	Phenotype
*AGK*	610345	AR	614691	Cataract 38, congenital cataract	Congenital cataract
*BFSP1*	603307	AD/AR	611391	Cataract 33, multiple types	Cortical, nuclear, punctate lamellar cataracts
*BFSP2*	603212	AD	611597	Cataract 12, multiple types	Lamellar, cortical, nuclear embryonic, ‘scattered lens opacities’
*CHMP4B*	610897	AD	605387	Cataract 31, multiple types	Posterior subcapsular cataract, progressing to affect nucleus and anterior subcapsular regions. Posterior polar cataracts
*CRYAA*	123580	AD/AR	604219	Congenital cataract; cataract 9, multiple types	Nuclear, zonular, central nuclear, laminar, lamellar, anterior polar, posterior polar, cortical, embryonal, anterior subcapsular, fan-shaped, total cataracts
*CRYAB*	123590	AD/AR	613763	Cataract 16, multiple types	Nuclear, posterior polar, nuclear, lamellar cataracts
*CRYBA1*	123610	AD	600881	Cataract 10, multiple types; cataract congenital zonular with sutural opacities	Cataract 10, cataract congenital zonular with sutural opacities, nuclear, lamellar cataract
*CRYBA2*	600836	AD	115900	Cataract 42	Multifocal cataract, congenital, juvenile, posterior polar, early cataract
*CRYBA4*	123631	AD	610425	Cataract 23	Lamellar and nuclear cataracts
*CRYBB1*	600929	AD/AR	611544	Congenital nuclear cataract; cataract 17, multiple types	Congenital nuclear cataract, pulverulent cataracts
*CRYBB2*	123620	AD	601547	Cataract 3, multiple types	Coppock-like cataract, cerulean cataracts
*CRYBB3*	123630	AD/AR	609741	Congenital nuclear cataract; Cataract 22	Congenital, cortical, nuclear cataract
*CRYGB*	123670	AD	615188	Cataract 39, multiple types	Anterior polar, lamellar cataract
*CRYGC*	123680	AD	604307	Cataract 2, multiple types; AD cataract coppock-like;	Cataract, variable zonular pulverulent, coppock-like cataract
*CRYGD*	123690	AD	115700	Cataracts; cataract 4, multiple types, congenital cerulean cataract	Aculeiform, progressive, congenital cerulean cataract
*CRYGS*	123730	AD	116100	Cataract 20, multiple types	Progressive polymorphic cortical cataract, progressive ‘opalescent’ cataract
*CYP51A1*	601637	AR	N/A	Autosomal recessive cataract due to abnormal sterol metabolism	Not otherwise specified, congenital cataract total white cataracts
*DNMBP*	611282	AR	618415	Cataract 48	Infantile onset cataracts
*EPHA2*	176946	AD	116600	Age-related cortical cataract; cataract 6, multiple types	Age-related cortical cataract. Persistent foetal vasculature, posterior polar, posterior subcapsular cataracts
*FOXE3*	601094	AD/AR	612968	Autosomal dominant cataracts; cataract 34, multiple types.	Membranous, posterior subcapsular cataracts
*FYCO1*	607182	AR	610019	Congenital cataract; cataract 18	Nuclear cataract, congenital cataracts
*GJA3*	121015	AD	601885	Zonular pulverulent cataract; cataract 14, multiple types	Nuclear pulverulent, zonular pulverulent cataract posterior polar, nuclear coralliform, embryonal nuclear, coppock-like cataracts
*GJA8*	600897	AD	116200	Cataract 1, multiple types	Zonular pulverulent, nuclear progressive
*HSF4*	602438	AD/AR	116800	Cataracts; cataract 5, multiple types	Lamellar, zonular stellate, anterior polar cataracts
*LEMD2*	616312	AR	212500	Cataract 46, juvenile-onset	Juvenile-onset ‘Hutterite-type cataract’
*LIM2*	154045	AR	615277	Cortical pulverulent cataract; Cataract 19	Cortical pulverulent cataract, congenital total cataract, nuclear cataract
*LSS*	600909	AR	616509	Cataract 44	Total cataract
*MAF*	177075	AD	610202	Cataract, pulverulent or cerulean, without microcornea; cataract 21, multiple types	Cataract, pulverulent or cerulean, without microcornea, cataract 21, cortical pulverulent, nuclear pulverulent with progression to posterior subcapsular cataract. Lamellar, anterior polar, nuclear, anterior subcapsular cataracts
*MIP*	154050	AD	615274	Cataracts; cataract 15, multiple types	Cataract 15, anterior and posterior polar, cortical cataract, progressive punctate lamellar, non-progressive congenital lamellar and sutural cataracts, embryonal nuclear cataracts
*NHS*	300457	XD	302200	Cataract 40, X-linked	Total nuclear, sutural, lamellar, zonular, perinuclear, posterior stellate cataracts
*PITX3*	602669	AD, AR	610623	Cataract 11, multiple types	Cataract 11, posterior polar, total and cortical cataracts
*TDRD7*	611258	AR	613887	Cataract 36	Congenital cataracts
*UNC45B*	611220	AD	616279	?Cataract 43	Posterior subcapsular and central cataracts
*VIM*	193060	AD	116300	Cataract 30, pulverulent	Pulverulent cataracts
*WFS1*	606201	AD/AR	116400	?Cataract 41	Congenital nuclear cataract

AD, Autosomal dominant; AR, autosomal recessive; XD, x-linked dominant.

##### Cytoskeletal structural proteins

Beaded filament structural proteins (BFSPs) are a type of intermediate filament protein that are expressed in various cells of the body, such as epithelial, glial and muscle cells. *BFSP1* and *BFSP2* are genes encoding Filensin and Phakinin, respectively, which are proteins expressed exclusively in the lens. They are intimately associated with crystallins, where they form part of a highly organised cytoskeleton and are also thought to have an important role in lens development and differentiation.^20^ Autosomal recessive *BFSP2* mutations cause cataract, but interestingly it has been suggested that heterozygous carriers of these mutations may be predisposed to develop age-related cataract and myopia (Table 2).^20^

**Table 2.** Genetic mutations associated with cataract and other ocular malformations. Detailed phenotype data sourced from Genomics England PanelApp and OMIM cataract databases and associated relevant publications.

Gene symbol	Gene/locus MIM number	Inheritance	Disease MIM number	Disease name	Ocular comorbidities (besides cataract)
*CRYBA1*	123610	AD	600881	Cataract 10, multiple types	Nystagmus, esotropia
*CRYBA2*	600836	AD	115900	Cataract 42	Eccentric pupil, glaucoma, myopia
*CRYBA4*	123631	AD/AR	610425	Cataract 23	Microcornea, microphthalmia
*CRYBB1*	600929	AD/AR	611544	–	Microcornea, nystagmus, iris and choroid coloboma
*CRYBB2*	123620	AD	601547	Cataract 3, multiple types	Microphthalmia, microcornea, coloboma (posterior segment), glaucoma
*CRYGC*	123680	AD	604307	Cataract 2, multiple types; AD cataract coppock-like	Microcornea
*CRYGD*	123690	AD	115700	Cataract 4, multiple types	Microcornea
*DNMBP*	611282	AR	618415	Cataract 48	Nystagmus, amblyopia, exotropia
*EPHA2*	176946	AD	116600	Cataract 6, multiple types	Microcornea
*FOXE3*	601094	AD/AR	610256	Peters anomaly	ASD, ocular dysgenesis, corneal opacity, Peters anomaly, microphthalmia, microcornea, nystagmus
*GJA3*	121015	AD	601885	Cataract 14, multiple types	Microphthalmia, microcornea
*GJA8*	600897	AD	116200	Cataract-microcornea syndrome	Microcornea, microphthalmia, glaucoma, ASD, coloboma, sclerocornea, total corneal opacification
*MAF*	177075	AD	610202	–	Microcornea, iris coloboma, microcornea, glaucoma, microphthalmia, myopia, nystagmus, Peters anomaly
*NHS*	300457	XD	302200	Cataract 40, X-linked	Microcornea, microphthalmia
*OPA3*	258501	AD	258501	Autosomal dominant optic atrophy with cataract (ADOAC)	Optic atrophy
*P3H2*	610341	AR	614292	–	High myopia, lens dislocation, retinal detachment, vitreoretinal degeneration
*PAX6*	607108	AD	106210	–	Cataract with late-onset corneal dystrophy
*PITX3*	602669	AD	610623	–	Anterior segment dysgenesis
*PXDN*	605158	AR	269400	–	Corneal opacity, developmental glaucoma; corneal opacification associated with other ocular anomalies (COPA)
*VSX2*	142993	AR	610092	–	Microphthalmia and iris abnormalities. Microphthalmia with coloboma type 3, microphthalmia isolated type 2

AD, Autosomal dominant; AR, autosomal recessive; XD, x-linked dominant; ASD, anterior segment dysgenesis.

##### Membrane proteins

The lens is an avascular structure and therefore membrane proteins have an important role in maintenance and metabolic homeostasis. Genetic mutations in over 10 membrane protein genes lead to inappropriate transport of ions, solutes and water between cells in the human lens (Table 1). Gap junction channels are made up of two connexons composed of six subunits called connexins.^21^ Connexins have an important role in lens microcirculation, particularly in the supply of metabolites and nutrients towards the centre of the lens, and outward flow of unwanted ions and by-products to the periphery.^22^ They are able to form functional hemichannels in a variety of species. Three particular cataract-causing mutations result in increased hemichannel activity: *GJA3* (encoding human lens connexin 46) c.427G>A, p.(Gly143Arg) and c.56C>T, p.(Thr19Met), and *GJA8* (encoding connexin 50) c.137G>T, p. (Gly46Val). Two mutations in *GJA8* c.827C,T, p.(Ser276Phe) and c.131T>C, p.(Val44Ala) decrease hemichannel activity and have also been implicated in the formation of cataract.^23^ Mutations in these genes are most commonly associated with zonular and nuclear pulverulent cataracts with varying progression.^23–25^ These mutations are most frequently missense, and *GJA8* is also associated with microcornea.^26,27^

Mutations in genes encoding major intrinsic proteins account for 5% of all inherited cataract.^28^ Aquaporin-0 is the most expressed membrane protein in the human lens and variants are inherited in an autosomal dominant pattern producing a variety of cataract phenotypes (Table 1).^29,30^ It functions primarily as a water channel, but recent research in mouse models suggests that it may also modulate gap junctions in the presence of BFSPs.^31^ Lens intrinsic membrane protein-2 (*LIM2*) is the gene responsible for a membrane protein with four transmembrane domains called MP19, which contribute to lens transparency.^32–34^ Three *LIM2* missense mutations have been described that cause autosomal recessive cataract (1) c.313T>G, p.(Phe105Val) mutation associated with cortical cataract; (2) c.587G>A, p.(Gly154Glu) mutation in a family causing autosomal recessive cataract; and (3) c.233G>A, p.(Gly78Asp) mutation in a consanguineous Pakistani family with nuclear cataracts.^35,36^

Other proteins working at the membrane include EPHA2 and DNMBP. *EPHA2* encodes a membrane-bound protein tyrosine kinase,^37^ and mutations have been shown to account for approximately 5% of inherited cataracts in the Australian population.^38^ Most known causative mutations occur in the sterile-α-motif region of the molecule that affects the structure of the EPHA2 protein and impairs cell migration in human and mouse fibroblast lens epithelial cells.^39^ Mislocalisation of two mutant proteins away from the cell membrane has also been implicated in cataractogenesis.^37^ *EPHA2* mutations are most often associated with autosomal dominant posterior polar cataract. *DNMBP* encodes a protein that regulates the configurations of cell junctions through binding to tight junction protein 1. Biallelic loss-of-function variants have been shown to result in autosomal recessive cataract with other ocular features, including pupil abnormalities, strabismus and nystagmus.^40^

##### Transcription factors

Heat shock transcription factor 4 (HSF4) protects lens proteins from cell stressors and has a regulatory role in the differentiation of lens fibre cells.^41^ Mutations in *HSF4* most often produce lamellar cataract, which can be present at birth or develop in early childhood, and can be inherited in a dominant or recessive mode. Of the 16 mutations described so far, 13 are missense and 11 occur in the protein’s DNA binding domain; these are autosomal dominant, suggesting this region is essential for normal protein function.^42^

Similarly, *MAF* encodes a transcription factor containing the basic-leucine zipper (bZIP) domain in which 7 of the 18 known mutations are associated with ocular defects such as iris coloboma, glaucoma, microcornea, microphthalmia, myopia, nystagmus and Peters anomaly.^42^ This suggests the bZIP domain has a critical role in eye development. Mutations in the N-terminal upstream of a transactivation domain encoding region of the *MAF* gene are associated with Ayme-Gripp syndrome (cataract, reduced growth, sensorineural hearing loss, learning disability, brachycephaly and seizures) and Asperger syndrome (Table 3).^42^

**Table 3.** Genetic mutations associated with syndromic cataract. Detailed phenotype data from Genomics England PanelApp and OMIM cataract databases and associated relevant publications.

Gene symbol	Gene/locus MIM number	Inheritance	Disease MIM number	Disease name	Frequent associated systemic features
*ADAMTS10*	608990	AR	277600	Weill–Marchesani syndrome	Short stature, brachycephaly, joint stiffness
*AGK*	610345	AR	212350	Sengers syndrome	Hypertrophic cardiomyopathy, skeletal myopathy, exercise intolerance
*AGPS*	603051	AR	600121	Rhizomelic chondrodysplasia punctata type 3	Short stature, broad nasal bridge, epicanthus, high-arched palate, dysplastic external ears, micrognathia, congenital contractures, dwarfism, severe mental disability with spasticity
*ALDH18A1*	138250	AD/AR	616603	Autosomal dominant/recessive cutis laxa-3 (ADCL3/ARCL3)	Mental disability, joint hypermobility, skin hyperelasticity, metabolic abnormalities: hyperammonemia/prolinemia/ornithinemia
*B3GLCT*	610308	AR	261540	Peters-plus syndrome	Peters anomaly, growth retardation, short stature, brachydactyly, developmental delay
*BCOR*	300166	XD	300485	Oculofaciocardiodental syndrome	Eye anomalies (microphthalmia, glaucoma), facial abnormalities (long narrow face, high nasal bridge, pointed nose, cleft palate), cardiac anomalies(atrial/ventricular septal defect, floppy mitral valve), dental abnormalities (canine radiculomegaly, delayed dentition, oligodontia, etc.).
*COL2A1*	120140	AD	609508	Stickler syndrome type I non-syndromic ocular	Congenital vitreous abnormality (vestigial gel in retrolental space) with midline clefting, flat midface, hearing loss, mild spondyloepiphyseal dysplasia, early onset arthritis
*COL4A1*	120130	AD	175780	–	Brain small vessel disease with or without ocular anomalies; microphthalmia
*COL4A5*	303630	XD	301050	Alport syndrome	Progressive renal failure, hearing loss and lenticonus, corneal erosions, retinal flecks
*COL11A1*	120280	AD	154780	Marshall syndrome; Stickler syndrome	Hearing loss, retinal detachment, midfacial hypoplasia, palatal hypoplasia
*COL18A1*	120328	AR	267750	Knobloch syndrome	High myopia, vitreoretinal degeneration, retinal detachment
*CRYAB*	123590	AD/AR	613763	–	Myofibrillar myopathy, adult onset cardiomyopathy, dilated cardiomyopathy
*CYP27A1*	606530	AR	213700	Cerebrotendinous xanthomatosis	Childhood cholestasis, tendon xanthomas. Neurological complications in adulthood including neuropsychiatric disturbance, increased muscle tone, ataxia, dystonia, seizures
*CYP51A1*	601637	AR	–	–	Developmental delay, spastic diplegia, and cryptogenic neonatal liver cirrhosis, neonatal cholestatic jaundice
*DCR*	–	IC	190685	Down’s syndrome	Mental disability, characteristic facies, cardiac anomalies, gastrointestinal tract disorders, leukaemia, hearing loss, early Alzheimer’s disease
*DHCR7*	602858	AR	270400	Smith–Lemli–Opitz syndrome	Hypotonia, microcephaly, micrognathia, craniofacial abnormalities, postaxial polydactyly, syndactyly, hypospadias, developmental delay
*DMPK*	605377	AD	160900	Myotonic dystrophy 1	Myotonia muscular dystrophy, cataracts, hypogonadism, frontal balding, ECG changes
*EED*	605984	AD	617561	Cohen–Gibson syndrome	Dysmorphic facial features, advanced bone age, skeletal anomalies, large hands, long fingers and camptodactyly, scoliosis and cervical spinal abnormalities
*EIF2B2*	606454	AR	603896	Leukoencephalopathy with vanishing white matter	Variable neurological features, including progressive cerebellar ataxia, spasticity, cognitive impairment associated with white matter lesions on brain imaging
*EPHA2*	176946	AD	116600	–	Microcornea, phacodenesis, neurodevelopmental delay, mild dysmorphic features
*ERCC2*	126340	AR	610756	Cerebrooculofacioskeletal syndrome (COFS2); trichothiodystrophy 1(TTD1)	COFS2: microcephaly, prominent nose, microphthalmia, blepharophimosis, large ears, overlapping upper lip, long philtrum, micrognathia. TTD1: brittle sulphur-deficient hair, cutaneous, neurologic and growth abnormalities
*ERCC3*	133510	AR	616390	Trichothiodystrophy 2(TTD2)	Brittle sulphur-deficient hair, cutaneous, neurologic and growth abnormalities
*ERCC6*	609413	AR	133540	Cockayne syndrome type B(CSB); cerebrooculofacioskeletalsyndrome 1(COFS1)	CSB: progeroid appearance, cachectic dwarfism, failure to thrive, mental disability, loss of adipose tissue, joint contractures, sensorineural hearing loss. COFS1: microcephaly, mental disability, facial dysmorphism, arthrogyrposis
*ERCC8*	609412	AR	216400	Cockayne syndrome type A MIMID	Progeroid appearance, cachectic dwarfism, mental disability, loss of adipose tissue, sensorineural hearing loss.
*FAM126A*	610531	AR	610532	Leukodystrophy hypomyelinating 5	Hypomyelination resulting in psychomotor regression, mental disability, sensorimotor peripheral neuropathy
*FBN1*		AD		Marfan syndrome	Skeletal, ocular and cardiovascular abnormalities. Long limbs and fingers, high-arched palate, aortic dilatation/regurgitation, myopia, subluxation of lens
*FOXE3*	601094	AD	612968	Cataract 34, multiple types	Vitreoretinal dysplasia, neurodevelopmental delay, joint laxity
*FTL*	134790	AD	600886	Hyperferritinemia-cataract syndrome	Hyperferritinemia, microcytic anaemia
*GALK1*	604313	AR	230200	Galactokinase deficiency with cataracts	Elevated plasma galactose with milder features of galactosaemia
*GALT*	606999	AR	230400	Galactosemia	Elevated plasma galactose with poor feeding with poor weight gain, vomiting and diarrhoea, liver cell damage and lethargy
*GCNT2*	600429	AR	116700	Adult i blood group with congenital cataract	Adult i blood group
*GEMIN4*	606969	AR	617913	–	Neurodevelopmental disorder with microcephaly, renal abnormalities
*GNPAT*	602744	AR	222765	Rhizomelic chondrodysplasia punctata type 2	Rhizomelic skeletal dysplasia, mental disability
*GTF2H5*	608780	AR	616395	Trichothiodystrophy 3, photosensitive	Brittle sulphur-deficient hair, cutaneous, neurologic and growth abnormalities
*HMX1*	142992	AR	612109	Oculoauricular syndrome	Microphthalmia, microcornea, corneal opacity, coloboma, external ear abnormalities
*HTRA2*	606441	AR	617248	3-methylglutaconic aciduria, type VIII	Death in infancy. Hypotonia, abnormal movements, respiratory insufficiency, lack of development with seizures
*INPP5K*	607875	AR	617404	Congenital muscular dystrophy with cataracts and mild cognitive impairment (MDCCAID)	Muscular dystrophy with progressive muscle weakness in childhood and mild cognitive impairment
*JAM3*	606871	AR	613730	–	Haemorrhagic destruction of the brain, subependymal calcification
*LONP1*	605490	AR	600373	CODAS syndrome (cerebral, ocular, dental, auricular, skeletal)	Developmental delay, craniofacial abnormalities, ptosis, median nasal groove, delayed tooth eruption, anomalous cusp morphology, malformed helices, hearing loss, short stature, delayed epiphyseal ossification, metaphyseal hip dysplasia, vertebral coronal clefts
*LSS*	616509	AR	600909	–	Congenital cataract, hypotrichosis.
*MAF*	177075	AD	610202	Ayme-Gripp syndrome	Sensorineural hearing loss, intellectual disability, seizures, brachycephaly, a distinctive flat facial appearance and reduced growth
*MAN2B1*	609458	AR	248500	Mannosidosis alpha types I and II	Progressive mental disability, immune deficiency, impaired hearing and Hurler-like skeletal changes
*MSMO1*	607545	AR	616834	–	Microcephaly, psoriasiform dermatitis
*MYH9*	160775	AD	155100	Epstein syndrome; Fechtner syndrome	Nephritis, mild hearing loss, thrombocytopaenia
*NDP*	300658	XR	310600	Norrie disease	Very early childhood blindness due to degenerative and proliferative changes of the neuroretina with or without progressive mental disability and psychosis and deafness
*NF2*	607379	AD	101000	Neurofibromatosis type 2	Multiple neoplasia syndrome. Tumours of either cranial nerve (usually bilateral), meningiomas and schwannomas
*NHS*	300457	XD	302200	Nance–Horan syndrome	Microcornea, dental anomalies for example, Hutchinsonian incisors, mesiodens, dysmorphic features for example, large anteverted pinna, mental disability
*OCRL*	300535	XR	309000	Lowe syndrome	Hydrophthalmia, mental disability, vitamin D-resistant rickets, amino aciduria, reduced ammonia produced by kidney
*PEX1*	602136	AR	214100	Peroxisome biogenesis disorder 1A (Zellweger) (ZS) Refsum disease infantile; Adrenoleukodystrophy neonatal	Neurological abnormalities, characteristic dysmorphism and hepatomegaly, death in infancy. Symptoms similar to ZS with survival to early childhood. Symptoms similar to ZS with longest survival 3–11 years of age
*PEX2*	170993	AR	614866	Peroxisome biogenesis disorder 5A, (Zellweger)	Neurological abnormalities, characteristic dysmorphism and hepatomegaly, death in infancy
*PEX3*	603164	AR	614882	Peroxisome biogenesis disorder 10A (Zellweger)	Neurological abnormalities, characteristic dysmorphism and hepatomegaly, death in infancy
*PEX5*	600414	AR	214110/202370/616716	Peroxisome biogenesis disorder 2A (Zellweger) Peroxisome biogenesis disorder 2B Rhizomelic chondrodysplasia punctata, type 5	Neurological abnormalities, characteristic dysmorphism and hepatomegaly, death in infancy. Rhizomelic skeletal dysplasia, mental disability
*PEX6*	601498	AR	614862	Peroxisome biogenesis disorder 4A (Zellweger)	Neurological abnormalities, characteristic dysmorphism and hepatomegaly, death in infancy
*PEX7*	601757	AR	215100	Rhizomelic chondrodysplasia punctata type 1; Refsum disease; peroxisome biogenesis disorder	Rhizomelic skeletal dysplasia, mental disability. Symptoms similar to ZS with survival to early childhood. Neurological abnormalities, characteristic dysmorphism and hepatomegaly, death in infancy
*PEX10*	602859	AR	614870	Peroxisome biogenesis disorder 6A (Zellweger); adrenoleukodystrophy neonatal	Neurological abnormalities, characteristic dysmorphism and hepatomegaly, death in infancy. Symptoms similar to ZS with longest survival 3–11 years of age
*PEX11B*	603867	AR	614920	Peroxisome biogenesis disorder	Neurological abnormalities, characteristic dysmorphism and hepatomegaly, death in infancy
*PEX12*	614859	AR	614859	Peroxisome biogenesis disorder 3A (Zellweger); peroxisome biogenesis disorder complementation group 3	Neurological abnormalities, characteristic dysmorphism and hepatomegaly, death in infancy
*PEX13*	601789	AR	614883	Peroxisome biogenesis disorder 11A (Zellweger); adrenoleukodystrophy neonatal	Neurological abnormalities, characteristic dysmorphism and hepatomegaly, death in infancy. Symptoms similar to ZS with longest survival 3–11 years of age
*PEX14*	601791	AR	614887	Peroxisome biogenesis disorder 13A	Neurological abnormalities, characteristic dysmorphism and hepatomegaly, death in infancy
*PEX16*	603360	AR	614876	Peroxisome biogenesis disorder 8A (Zellweger);	Neurological abnormalities, characteristic dysmorphism and hepatomegaly, death in infancy
*PEX19*	600279	AR	614886	peroxisome biogenesis disorder 12A (Zellweger)	Neurological abnormalities, characteristic dysmorphism and hepatomegaly, death in infancy
*PEX26*	608666	AR	614872	Peroxisome biogenesis disorder 7A (Zellweger) (ZS); Refsum disease infantile; adrenoleukodystrophy neonatal	Neurological abnormalities, characteristic dysmorphism and hepatomegaly, death in infancy. Symptoms similar to ZS with survival to early childhood. Symptoms similar to ZS with longest survival 3–11 years of age
*PITX3*	602669	AD, AR	610623	–	Mental disability, choreiform movements, increased muscle tone, deep tendon reflexes of lower extremities
*POMT1*	607423	AR	236670	Walker–Warburg syndrome	Cobblestone (type II) lissencephaly, cerebellar malformations, retinal malformations
*RAB18*	602207	AR	614222	Warburg micro syndrome 3	Congenital microcephaly, cortical dysplasia, microcornea, optic atrophy, severe mental disability, hypotonic diplegia, hypogenitalism
*RAB3GAP1*	602536	AR	600118	Warburg micro syndrome 1	Congenital microcephaly, cortical dysplasia, microcornea, optic atrophy, severe mental disability, hypotonic diplegia, hypogenitalism.
*RAB3GAP2*	609275	AR	212720	Warburg micro syndrome 2 (WMS2); Martsolf syndrome	Congenital microcephaly, cortical dysplasia, microcornea, optic atrophy, severe mental disability, hypotonic diplegia, hypogenitalism. Milder phenotype of WMS2. Microphthalmia, postnatal microcephaly, developmental delay
*RECQL4*	603780	AR	268400	Rothmund–Thompson syndrome, type 2	Poikiloderma, congenital bone defects, increased risk of osteosarcoma in childhood, skin cancer in adulthood
*SC5D*	602286	AR	607330	Lathosterolosis	Facial dysmorphisms, severe microcephaly, micrognathia, neonatal jaundice, mental disability, hexadactyly, syndactyly, liver disease
*SEC23A*	610511	AR	607812	Craniolenticulosutural dysplasia	Facial dysmorphism, late closing fontanelle, skeletal defects
*SIL1*	608005	AR	248800	Marinesco–Sjogren syndrome	Cerebellar ataxia, progressive myopathy
*SLC2A1*	138140	AD	608885	Stomatin-deficient cryohydrocytosis with neurologic defects	Delayed psychomotor development, seizures, pseudohyperkalaemia
*SLC33A1*	603690	AR	614482	–	Congenital cataracts, hearing loss, and neurodegeneration
*SRD5A3*	611715	AR	612713	Kahrizi syndrome	Mental disability, coloboma, kyphosis
*TFAP2A*	107580	AD	113620	Branchiooculofacial syndrome	High-arched palate, prominent philtrum, narrow ear canals, abnormal pinnae, periorbital and scalp cysts
*WRN*	604611	AR	277700	Werner syndrome	Accelerated ageing appearance and disorders associated with aging including diabetes mellitus, osteoporosis, premature atherosclerosis, benign and malignant neoplasms
*XYLT2*	608125	AR	605822	Spondyloocular syndrome	Bone fragility, hearing defects, retinal detachment, facial dysmorphism, immobile spine, platyspondyly

AD, Autosomal dominant; AR, autosomal recessive; XD, x-linked dominant; XR, x-linked recessive; IC, isolated cases.

*FOXE3* is one of a family of genes encoding transcription factors with a ‘forkhead’ DNA binding domain and has a role in lens embryology. Twenty-four unique mutations have been shown to affect various ocular structures, including cornea, iris, lens and retina (Table 2).^42^ Most mutations are missense and 10 occur in the forkhead DNA binding domain, resulting in defective or non-functional protein. Pituitary homeobox 3 (PITX3) proteins are transcription factors that have been shown to play an important role in controlling lens epithelial cell maintenance and fibre cell differentiation in mice.^43^ They contain the homeobox and the otp, aristaless, rax (OAR) domains; however, surprisingly no cataract-causing mutations have been described in either domain. Mutations in *PITX3* genes most often cause posterior polar cataract with or without anterior segment dysgenesis (ASD), but can result in other ocular defects such as corneal opacity, microphthalmia, microcornea, nystagmus and glaucoma.^42^

#### Syndromic congenital cataract

Syndromic congenital cataract manifests as a result of various types of mutations, including chromosomal abnormalities, loss-of-heterozygosity, mitochondrial disorders, triplet repeat disorders and more complex genetic disorders such as diabetes.^35^ Cataracts show phenotypic variability and are associated with a myriad of systemic dysmorphic features.^44^

Chromosomal abnormalities featuring cataract include those with an extra copy of chromosome 21 and 13 causing Down’s syndrome (trisomy 21) and Patau syndrome (trisomy 13) respectively. Children with trisomy 21 are born with characteristic features and are prone to various health problems (Table 3), including ocular abnormalities such as cataract, upslanting palpebral fissures, astigmatism, iris abnormalities, strabismus, lacrimal system obstruction, blepharitis, retinal abnormalities, hypermetropia, amblyopia, nystagmus and myopia.^45^ Cataract has been found in 15% of cases of children with trisomy 21.^46^ Children with trisomy 13 commonly present with cataract as well as microphthalmia and ocular coloboma; however, they rarely live more than a few days or weeks due to multiple severe congenital abnormalities involving the craniofacial, musculoskeletal, cardiac, abdominal and nervous system.^47^

Multiple X-linked disorders demonstrate congenital cataracts (Table 3). Lowe syndrome is X-linked recessive, characterised by a triad of features including dense congenital cataracts, intellectual disability and proximal tubular dysfunction. It is caused by mutations in the *OCRL* gene that encodes inositol polyphosphate 5-phosphatase OCRL-1 and is involved in a large spectrum of intracellular processes, some of which affect actin stress fibres, polymerisation and distribution, which are likely to be responsible for the clinical phenotype.^48^ In the neonatal period, hypotonia and cataracts may be seen but the condition may not be diagnosed until later, when renal dysfunction becomes apparent.^49^

Norrie disease is an X-linked recessive disorder caused by mutations in the *NDP* gene, which encodes Norrin. There are genotype–phenotype correlations with missense mutations in exon 3 resulting in less severe phenotypes than nonsense mutations.^50^ Male children often present in the first few weeks of life with blindness due to bilateral retrolental vascularised masses with normal-sized eyes. Shallow anterior chamber, iris atrophy, cataracts and corneal opacity are common features, with eyes becoming phthisical over time.^51^

Male children with inherited Nance–Horan syndrome have typical facial dysmorphic features (long face, large ears, prominent nose) as well as dental and ocular abnormalities including cataract and developmental delay (Table 3).^52^ Interestingly, female carrier children may demonstrate a milder phenotype with Y-sutural lens opacities with cortical ‘riders’ and milder facial and dental abnormalities.^53^ Loss-of-function mutations in the *NHS* gene result in the syndromic disease; however, translocations such as duplications can cause non-syndromic isolated cataract.^52^ It has been suggested that this protein could be involved in the regulation of tight junction proteins or in the regulation of actin remodelling and maintaining cell morphology, but the function of this protein remains unknown.^54,55^

Myotonic dystrophy 1 is also inherited autosomal dominantly but occurs as a result of a cytosine–thymine–guanine (CTG) repeat disorder and is associated with a variety of ocular signs such as cataract, retinal degeneration, low intraocular pressure, eyelid ptosis, epiphora, corneal lesions, extraocular myotonia and weakness and abnormal central control of eye movement.^56^ Cataracts are often non-specific and appear as punctate opacities.^57^ Neurofibromatosis type 2 is caused by autosomal dominantly expressed mutations in the *NF2* gene. It classically presents in adulthood with bilateral vestibular schwannoma (VS); however, in children it most often presents with small posterior capsular or cortical edge cataracts as well as non-VS neurological signs and subtle skin tumours.^58^

Metabolic disorders are an important differential in the cause of congenital cataract, and these are often inherited in an autosomal recessive pattern. Systemic presentations may be mild with cataract being the initial sign. Zellweger spectrum disorder (ZSD) is an autosomal recessive metabolic peroxisome biogenesis disorder presenting with severe features in childhood that result in early death (Table 3); milder phenotypes include neonatal adrenoleukodystrophy and infantile Refsum disease.^59^ It is caused by mutations in *PEX* genes that encode proteins required for peroxisome biogenesis.^60^

Several rare autosomal recessive conditions are associated with the development of cataract. Rothmund–Thompson syndrome is a rare disorder with only 300 known cases. Two-thirds of cases are caused by a mutation in the *RECOL4* gene that is involved in DNA replication and repair, thus leading to widespread changes in skin, connective tissue and bone. There is an increased incidence of cancer in childhood and adolescence, in particular bone malignancies (osteosarcomas) and skin cancers, including squamous and basal cell carcinomas. Cockayne syndrome is a rare autosomal recessive disorder presenting with varying severity. Genetic mutations in *ERCC6* or *ERCC8* affect the repair of mitochondria and DNA damage – for example, due to environmental stressors such as sunlight leading to photosensitivity.^61^ Mutations in the *CYP27A1* gene lead to an accumulation of cholesterol and cholestenol in tissues and cause cerebrotendinous xanthomatosis (CTX).^62^ Developmental cataract is often an early ocular sign of this condition, along with systemic features such as infantile diarrhoea and tendon xanthomas; however, often a diagnosis of CTX is not made until adulthood when patients present with a variety of progressive neurological features. Early recognition and treatment with daily chenodeoxycholic acid can halt or even reverse severe neurological complications and improve prognosis.^63^ Galactosaemia, a rare inherited disorder of galactose metabolism screened for at birth in the UK, is caused by mutations in genes that encode enzymes of galactose. Galactokinase deficiency and classic galactosaemia are caused by mutations in the genes of galactokinase (*GALK1*) and galactose-1-phosphate (*GALT*), respectively (Table 3). Nearly 200 mutations in the *GALT* gene have been found and presentation is often severe in the first few weeks of life as infants are exposed to breast/formula milk.^64^ Accumulation of galactitol in the lens causes a characteristic ‘oil droplet’ cataract. Anterior and posterior subcapsular cataract may also occur.^65^ Early recognition and initiation of a galactose-free diet can reverse lens clouding and prevent fatal systemic consequences.^66^ Galactokinase deficiency is seen as a mild form of galactosaemia and paediatric cataract is often the major clinical feature.^67^

### Acquired causes

#### Congenital infections

Rubella is the most common congenital infection causing cataract worldwide.^68^ Maternal rubella infection during pregnancy puts the foetus at risk of developing a clinical rubella syndrome comprising a triad of deafness, cataract and cardiac disease. Rubella infection continues to be a public health problem in countries that do not have adequate national immunisation programmes.^69,70^ Other infections causing cataract include toxoplasmosis, CMV and herpes simplex (HSV I and II). It is common practice to screen for these infections when investigating the cause of paediatric cataract using the TORCH screen, but many factors can affect the positivity of the test, including maternal antibodies in neonates.^71^ There is some debate over the use of screening in routine practice and a move towards using specific tests for individual cases based on maternal antenatal history, child comorbidities and vaccination status.^72^

#### Trauma

Traumatic cataract makes up a significant proportion of childhood cataract.^73^ It has different considerations in terms of its presentation and management compared to true congenital cataract due to its unilaterality and potential damage to other ocular structures, such as angle recession leading to secondary glaucoma. Surgical management can be more complex but with overall less dense amblyopia and better chance of visual rehabilitation.^74^ Common objects causing trauma vary globally, with bow and arrows being the most common cause of ocular trauma in India, followed by sticks, stones and thorns, which are more common in East Africa.^74,75^

### Other common causes of cataract in children

#### Uveitic cataract and steroid-related cataract

Anterior uveitis is defined as inflammation of the iris and ciliary body. Juvenile idiopathic arthritis (JIA) associated uveitis is the leading cause of ocular morbidity in paediatric patients with uveitis and is associated with HLAB27 positivity. JIA describes a group of arthridities presenting in childhood, and uveitis is an extra-articular feature present in 11–13% of patients. It is more prevalent in females, those with oligoarthritis and positive antinuclear antibodies. Cataract occurring in the context of anterior uveitis may occur as a result of disease-associated inflammation or the prolonged use of steroids in its management, giving rise to deprivation amblyopia and visual loss.^76,77^ Most commonly steroids produce a posterior subcapsular cataract, and although the precise mechanism is unknown, glucocorticoids are thought to have an important role in the transcription of genes in lens epithelial cells.^78^

#### Radiation cataract

Exposure to radiation can cause the development of cataract. Children undergoing radiotherapy for treatment of childhood cancers such as leukaemia, central nervous system tumours, lymphomas, kidney cancer, neuroblastoma, soft tissue and bone sarcomas are therefore at risk of developing cataract. At higher doses of radiation – that is, above 3 Gy – cataract develops more quickly; however, more recent evidence suggests it can occur even at lower doses of radiation of 0.5 Gy, with the likelihood following a linear dose response.^1^ Doses of radiation cannot easily be reduced in the treatment of cancer, but measures can be taken to protect the eye and lens in high-risk patients, such as accurate radiation dose estimates and shielding.^1^

## Management

### Paediatric cataract surgery

The management of cataract presenting in children is more complicated than that of adult patients. Primarily the risks of performing early surgery need to be balanced with facilitating the best possible visual development in children during an amblyogenic period. In children, cataract surgery with or without anterior capsulotomy, posterior capsulotomy and anterior vitrectomy are performed.^79^ Intraocular lens (IOL) insertion is another point of contention. Findings from 5-year follow-up studies suggest that for children younger than 2 years old, inserting an IOL often results in similar post-operative vision when compared with contact lens only, it does not protect against post-operative glaucoma and results in additional requirement for intraocular surgery.^80,81^ Therefore, the recommendation is that children are left aphakic, and certainly those less than 6 months of age, until further evidence is presented.^82^ Selecting an IOL is difficult as the eye is growing and power calculations are more challenging because of difficulty attaining accurate measurements in the child. It is recommended that cataract should be extracted at 6 weeks of age for unilateral cataract and between 6 and 8 weeks of age in bilateral cataract.^80^ Rigorous post-operative care in children and regular follow up are essential for good long-term visual outcomes. Younger children undergoing lensectomy have better visual outcomes but are at higher risk of glaucoma, but conversely children having surgery later are at higher risk of strabismus.^80^ Non-surgical management may be appropriate with dilating drops for partial cataracts to increase light entering the eye. In galactosaemia cases, modification of diet and enzyme replacement may cause cataracts to stop progressing or even regress.^83,84^

### Genetic testing

Genetic testing is a key investigation for congenital cataracts, and recent studies have shown molecular diagnostic rates between 50% and 90% for bilateral cases.^3,85^ Current practice involves using a targeted gene panel of cataract-related genes using next-generation sequencing technology, which has ⩾90% coverage. However, whole-genome sequencing (screening of all coding and non-coding intergenic and intragenic regions) has been estimated to increase diagnostics rates by a further 40%.^86^ Genetic testing has been found to expedite diagnosis and provide a personalised clinical management plan, streamlining care pathways for patients, supporting informed genetic counselling and making informed decisions with regards to family planning.^5^ Research focuses on integrating this evidence into clinical practice and establishing robust genotype–phenotype correlations to aid prognosis and to ensure systemic features are detected earlier to reduce comorbidities.^87,88^

### Multidisciplinary care

Multidisciplinary management is essential to ensure full investigation and appropriate care pathways are established with paediatricians’ input, to exclude metabolic disorders, congenital infections and other syndromic and potentially life-threatening causes, especially while awaiting the results of genetic investigations.

Children require ongoing care within eye services where they will be seen regularly by ophthalmologists, orthoptists and optometrists to monitor the health and visual potential of the eye, and to ensure that any other amblyopic stimuli is minimised. In the UK, children and young people who are left with moderate reduction in their vision are referred to specialist advisory services for children with visual impairment in order to ensure that advice and support can be given to parents and to the educational services.

#### Assessment and clinical examination/investigation

An approach to assessment of children with cataract includes obtaining a history of the pregnancy and birth, with a focus on enquiring about maternal illness, drugs and medications in pregnancy, birth weight and neonatal events including jaundice treatment and clinical course. Subsequent history will be guided by the age of presentation, including current or past medical illness with particular attention to features of failure to thrive, developmental concerns and a thorough systems review. Family history and genogram should be obtained and investigations tailored to the history, age of presentation, gender and physical examination.

With a mobile and changing population, screening for congenital viral infections must be considered as there will be a small percentage of mothers who are not immune to rubella and thus at risk of becoming infected and passing on the infection to their unborn babies. Genetic testing for bilateral cases should be considered, whether through ophthalmic genetic specialists or clinical geneticists.^72^

#### Vision, development and supporting families

Families of children with congenital cataract require information and support the first time they present to the eye clinic. Parents have concerns about their child’s prognosis and potential visual outcome, and how it will affect their future development and educational achievements. As children with sight impairment must optimise their vision, parents require guidance in the early years to know how to promote their child’s vision in order to maximise learning. Infants and children in the UK are referred to specialist teachers for children with visual impairment to provide crucial developmental guidance and support to families as the child moves through the toddler years into early nursery and statutory education. This support should go hand in hand with the paediatrician’s involvement, together with ophthalmology input with ongoing follow up in the children’s low-vision services.

### Conclusion and future directions

Congenital cataract is a widely phenotypic heterogeneous disease with genetic and environmental causes to consider. Surgical management is the mainstay treatment for the cataract itself, but the clinical challenge is finding the cause. Many children presenting to health services with cataract with or without systemic features remain without a diagnosis. Genomic advances have accelerated the discovery of new cataract-causing mutations, and conditions associated with cataract continue to be identified. The ‘CAT-MAP’ database provides a comprehensive list of over 350 human and mouse genes and loci.^35^ This includes at least 16 human loci where the underlying causative gene remains unknown, as well as a list of cataract genes shown to cause a cataract phenotype in mice. Although there are genotype–phenotype discrepancies between mice and humans, these lists suggest there are still many genes to be discovered. Recent research is directed towards providing high-quality evidence of the clinical utility of genetic testing to facilitate its provision and successful integration into clinical practice.^85,88,89^ Accurate diagnosis of inherited cataract is crucial for patients and their families as it facilitates individualised genetic counselling. It enables the multidisciplinary team to best support optimum child development during a critical period, empower families with a diagnosis and help them to plan for the future.
